# Spermidine Oxidation-Mediated Degeneration of Retinal Pigment Epithelium in Rats

**DOI:** 10.1155/2017/4128061

**Published:** 2017-03-07

**Authors:** Koji Ohashi, Masaaki Kageyama, Katsuhiko Shinomiya, Yukie Fujita-Koyama, Shin-ichiro Hirai, Osamu Katsuta, Masatsugu Nakamura

**Affiliations:** Global Research and Development, Santen Pharmaceutical Co. Ltd., Ikoma-shi, Nara, Japan

## Abstract

Retinal pigment epithelium (RPE) degeneration is a crucial event in dry age-related macular degeneration and gyrate atrophy. The polyamine spermidine has been shown to induce RPE cell death in vitro. The present study aimed to establish a novel in vivo model of spermidine-induced RPE degeneration and to determine whether spermidine-induced RPE cell death involves oxidative mechanisms. In this study, spermidine caused ARPE-19 cell death in a concentration-dependent manner. This effect was prevented by removal of serum from the culture medium or treatment with amine oxidase inhibitors, N-acetylcysteine (NAC), or aldehyde dehydrogenase (ALDH). Intravitreal injection of spermidine into rats significantly increased the permeability of the blood-retinal barrier and decreased the amplitudes of scotopic electroretinogram a- and b-waves. Histological analysis revealed that spermidine induced vacuolation, atrophy, and dropout of RPE cells, leading to the disruption of photoreceptor outer segments. Simultaneous intravitreal administration of NAC and ALDH with spermidine prominently inhibited the functional and morphological changes induced by spermidine. In conclusion, this study demonstrated that the intravitreal administration of spermidine induced RPE cell dysfunction and death followed by photoreceptor degeneration in rats. These effects of spermidine are thought to be mediated by oxidative stress and a toxic aldehyde generated during spermidine oxidation.

## 1. Introduction

The retinal pigment epithelium (RPE) is a monolayer of cells located between the sensory retina and the choroid. The RPE exerts a variety of important functions involved in maintaining sensory retina homeostasis, including the regulation of nutrient transport to the photoreceptors, phagocytosis of distal tips of rod outer segments, absorption of stray light, and secretion of growth factors [1]. RPE degeneration predisposes photoreceptor cells to secondary damage and death consequent to the loss of support from the RPE and thus causes vision-threatening diseases such as dry age-related macular degeneration (dry AMD) [2, 3] and gyrate atrophy with hyperornithinemia [4].

Previous studies have suggested that the RPE degeneration observed in dry AMD and gyrate atrophy is caused by various factors, including oxidative stress [5] and ornithine accumulation [6]. Several animal models of RPE degeneration, such as sodium iodate-induced mouse, rat, and rabbit models [7–9], the ornithine-induced rat model [10], and the ornithine delta-aminotransferase deficient mouse [11], have been established and used in studies of the mechanisms of dry AMD and gyrate atrophy. However, the precise mechanism(s) underlying the degeneration of RPE and photoreceptor cells in these diseases are still not fully understood, and currently there are no approved drugs for the treatment of these conditions. A novel in vivo model of RPE degeneration would be useful for the elucidation of these mechanisms.

Polyamines such as spermine, spermidine, and putrescine are metabolites of ornithine and ubiquitous cellular components [12]. These polyamines have been reported to regulate various functions of RPE cells, including proliferation [13] and migration [14]. However, a previous in vitro study found that excessive spermine and spermidine induced the death of bovine RPE cells, suggesting that polyamines might be involved in the RPE degeneration associated with gyrate atrophy [15]. Previous studies of other cell lines suggested that toxic metabolites, particularly hydrogen peroxide and the toxic aldehyde acrolein, which are generated during polyamine oxidation, are involved in polyamine-induced cell death [16–19]. Therefore, the intravitreal administration of spermidine in vivo may induce RPE degeneration via spermidine oxidation.

The aims of this study were to establish a novel in vivo model of RPE degeneration, using spermidine as an inducer, and to determine whether oxidative mechanisms were involved in spermidine-induced RPE cell death. To achieve these aims, we examined the effects of intravitreal spermidine administration on the function and histology of the rat sensory retina and RPE and examined the effects of various inhibitors of the polyamine oxidation pathway on spermidine-induced RPE cell death in vitro and in vivo. We selected an intravitreal injection as an administration route of spermidine in in vivo studies, because it may be a suitable way to deliver an adequately high concentration of spermidine to the retina.

## 2. Methods

### 2.1. Materials

ARPE-19 cells were purchased from ATCC (Manassas, VA, USA). DMEM/F12 was obtained from Nacalai Tesque (Kyoto, Japan). Fetal bovine serum (FBS) and penicillin-streptomycin were supplied by Thermo Fisher (Waltham, MA, USA). The CellTiter 96® Aqueous One Solution cell proliferation assay reagent (containing the tetrazolium compound MTS) was provided by Promega (Madison, WI, USA). Spermidine and spermine were purchased from Merck Millipore (Billerica, MA, USA). Aminoguanidine was provided by Cayman Chemical (Ann Arbor, MI, USA). Dulbecco's phosphate-buffered saline (DPBS), pentamidine, N-acetylcysteine (NAC), and aldehyde dehydrogenase (ALDH) were supplied by Sigma-Aldrich (St. Louis, MO, USA). Glutaraldehyde and formalin were obtained from Wako (Osaka, Japan). 0.5% Tropicamide, 0.5% phenylephrine hydrochloride (Mydrin-P®), 0.4% oxybuprocaine hydrochloride (Benoxil®), and 0.5% levofloxacin ophthalmic solution (Cravit®) were provided by Santen Pharmaceutical (Osaka, Japan). 10% Fluorescein (Fluorescite®) was purchased from Alcon Japan (Tokyo, Japan). Ten mg/mL Ketamine (Ketalar®) was supplied by Daiichi Sankyo (Tokyo, Japan). 2% Xylazine (Selactar®) was obtained from Bayer Health Care (Tokyo, Japan). Mouse monoclonal anti-acrolein antibody (5F6) was provided by NOF Corporation (Tokyo, Japan). Histofine Simple Stain Rat MAX-PO (MULTI) was purchased from Nichirei Biosciences Inc. (Tokyo, Japan). DAB substrate kit was supplied by Dako Japan (Tokyo, Japan).

### 2.2. Cell Culture

ARPE-19 cells were cultured in DMEM/F12 supplemented with 10% FBS, 100 U/mL penicillin, and 100 mg/mL streptomycin in a humidified atmosphere of 95% air and 5% CO_2_ at 37°C, as previously described [20, 21]. Passage numbers 23–36 were used for the experiments.

### 2.3. Assessment of Cell Viability

ARPE-19 cell viability was assessed via an MTS assay; for this purpose, the CellTiter 96 Aqueous One Solution cell proliferation assay was used according to the manufacturer's protocol. In brief, ARPE-19 cells were seeded at a density of 10^4^ cells per well into 96-well plates. Twenty-four hours after seeding, spermidine was added to the medium in the presence or absence of 10% FBS to obtain final medium concentrations of 100–500 *μ*M. Twenty-four hours after the addition of spermidine, Aqueous One solution reagent was added to the medium, followed by a two-hour incubation. Next, the intensity of each colorimetric reaction was measured at 490 nm using a microplate reader (Bio-Rad, Hercules, CA, USA). To examine the effects of aminoguanidine, pentamidine, NAC, or ALDH on spermidine-induced ARPE-19 cell death, these compounds were added simultaneously with spermidine.

### 2.4. Animals

Animal experiments were performed in accordance with the Association for Research in Vision and Ophthalmology (ARVO) Statement concerning the use of animals in ophthalmic and vision research. The experimental procedure was approved and monitored by the Animal Care and Use Committee of the Nara Research & Development Center, Santen Pharmaceutical Co., Ltd. Six to eight-week-old female Brown Norway rats were purchased from Charles River Japan (Yokohama, Japan). Rats were housed under a 12-hour light/12-hour dark cycle and provided with food and water ad libitum.

### 2.5. Intravitreal Administration

Rats were anesthetized by an intramuscular injection of ketamine (87 mg/kg) and xylazine (13 mg/kg). Pupils were dilated with 0.5% tropicamide and 0.5% phenylephrine hydrochloride. DPBS, spermidine (1, 2, and 3 mM dissolved in DPBS), or spermine (1.5 mM dissolved in DPBS), each in a total injection volume of 10 *μ*L, were administrated into the vitreous bodies of both eyes per animal using a microinjector (Hamilton, Reno, NV, USA) with the aid of a dissecting microscope. Next, 0.5% of levofloxacin ophthalmic solution was applied to the ocular surface to prevent infection. Eyes treated with DPBS were used as controls. To examine the effects of NAC and ALDH on spermidine-induced RPE degeneration, these drugs were coadministered with spermidine.

### 2.6. Vitreous Fluorophotometry

The permeability of the blood-retinal barrier (BRB) was evaluated using vitreous fluorophotometry, as previously described [21]. Rats were anesthetized as described above on days 1, 3, 7, 14, and 28 after the administration of either DPBS or spermidine (10, 20, or 30 nmol/eye). Next, fluorescein (10 mg/kg) was injected intravenously, and the pupils were dilated with 0.5% tropicamide and 0.5% phenylephrine hydrochloride. After fluorescein was allowed to circulate for 45 minutes, the concentrations of vitreous and plasma fluorescein were measured using a Fluorotron Master (OcuMetrics, Mountain View, CA, USA). The permeability of the BRB was calculated according to the following formula:

(1) $$\begin{array}{c} \text{Permeability}=\frac{\text{Vitreous fluorescein (ng/mL)}}{\text{Plasma fluorescein (ng/mL)}}. \end{array}$$

In experiments to evaluate the effects of NAC and ALDH, we only measured the concentration of vitreous fluorescein to evaluate BRB permeability because the intravitreal administration of spermidine did not affect the concentration of plasma fluorescein in the time course experiment.

### 2.7. Electroretinogram

Scotopic electroretinogram (ERG) data were recorded on days 2, 6, 13, and 27 after the administration of either DPBS or spermidine (10, 20, or 30 nmol/eye), as previously described [20, 21]. For these recordings, rats were dark-adapted for at least 30 min. All manipulations were done under dim red light. Rats were anesthetized as described above, and the pupils were dilated with 0.5% tropicamide and 0.5% phenylephrine hydrochloride. Corneal anesthesia was achieved using topical 0.4% oxybuprocaine hydrochloride. A platinum electrode was placed in contact with the cornea, and a reference electrode and ground electrode were positioned on the nose and tail, respectively. Responses to a 3000 cd/m^2^ white light flash (10 ms) were amplified, filtered, and recorded using a portable ERG&VFP LE-3000 device (Tomey, Nagoya, Japan). The a-wave amplitudes were measured from the baseline to the trough of the a-wave, and the b-wave was measured from the trough of the a-wave to the peak of the b-wave.

### 2.8. Histology

Rats were sacrificed by bleeding on days 1, 3, 7, 14, and 28 after the administration of either DPBS or spermidine (10, 20, or 30 nmol/eye). The eyes were enucleated and fixed in a mixture of 2.5% glutaraldehyde and 9% formalin for 2 hours, followed by 10% formalin overnight. Fixed eyes were embedded in paraffin. Three-micrometer-thick sections were cut with a microtome and stained with hematoxylin and eosin (HE). Photographs at the posterior region of retina were taken. In this experiment, we also examined the effect of spermine (15 nmol/eye), another polyamine, as reference.

### 2.9. Transmission Electron Microscopy

Rats were sacrificed by bleeding at 6 hours and 4 days after the administration of either DPBS or spermidine (20 nmol/eye). The eyes were enucleated and fixed in Karnovsky's fixative, postfixed in 1% osmium tetroxide for 2 hours at 4°C, dehydrated, and embedded in epoxy resin for electron microscopy. One-micrometer-thick sections were stained with toluidine blue and used to select suitable areas for electron microscopy. Finally, 80 nm ultrathin sections were cut, stained with uranyl acetate and lead citrate, and observed with an H-7600 transmission electron microscope (Hitachi High-Technologies, Tokyo, Japan).

### 2.10. Immunohistochemistry

Rats were sacrificed by bleeding on days 1 and 3 after the administration of either DPBS or spermidine (20 nmol/eye). The eyes were enucleated and fixed at 4°C for several days in phosphate buffer containing 4% formaldehyde. Fixed eyes were embedded in paraffin. Following deparaffinization and blocking, mouse monoclonal anti-acrolein antibody which is specific for acrolein-modified proteins and the secondary antibody Histofine Simple Stain Rat MAX-PO (MULTI) were sequentially applied to 4-micrometer-thick sections for 1 hour each, at room temperature. Specific immunoreactivity was visualized using a DAB substrate kit. Finally, counter staining was performed using 3% Giemsa staining solution.

### 2.11. Statistical Analysis

EXSAS Version 6.10 (Arm, Osaka, Japan) was used for the statistical analysis. Data are expressed as means ± standard errors of the means. Student's *t*-test or the Aspin–Welch *t*-test was used to compare two groups. A one-way analysis of variance (ANOVA), followed by Dunnett's multiple comparison test, was used to compare more than three groups. Differences were considered to be statistically significant if *P* < 0.05.

## 3. Results

### 3.1. Protective Effects of Polyamine Oxidation Pathway Inhibitors on Spermidine-Induced ARPE-19 Cell Death

**Figure 1:**
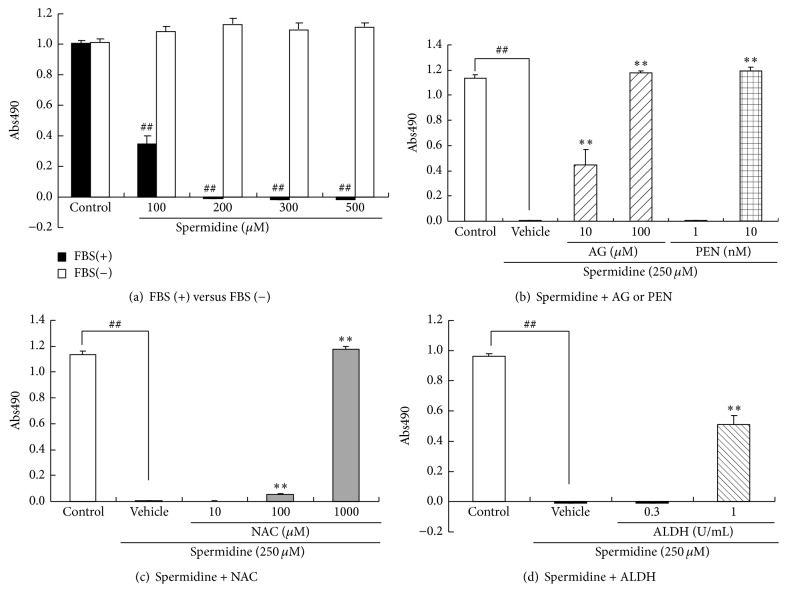
Effects of serum removal and treatment with amine oxidase inhibitors, an antioxidant compound, and aldehyde dehydrogenase (ALDH) on spermidine-induced ARPE-19 cell death. Cell viability was assessed with a MTS assay 24 h after the addition of spermidine. The effects of (a) serum removal and treatment with (b) amine oxidase inhibitors, (c) an antioxidant, and (d) ALDH when applied simultaneously with spermidine. Each column represents a mean ± standard error of the mean of four wells. ^##^*P* < 0.01 versus control. ^*∗∗*^*P* < 0.01 versus spermidine. AG: aminoguanidine. PEN: pentamidine.

First, we confirmed the concentration-dependent effect of spermidine on ARPE-19 cell viability and the serum-dependence of this effect. In the presence of 10% FBS, spermidine (100–500 *μ*M) induced ARPE-19 cell death in a concentration-dependent manner (Figure 1(a)). In contrast, in the absence of FBS, spermidine did not induce cell death (Figure 1(a)). These results suggest that a spermidine byproduct generated by serum components, but not spermidine itself, induced ARPE-19 cell death.

We next examined the effects of various polyamine oxidation pathway inhibitors on spermidine-induced ARPE-19 cell death. In the presence of 10% FBS, the amine oxidase inhibitors aminoguanidine (10–100 *μ*M) and pentamidine (0.01–0.1 *μ*M) inhibited spermidine (250 *μ*M)-induced cell death (Figure 1(b)). In addition, the antioxidant NAC (100–1000 *μ*M) and the aldehyde metabolizing enzyme ALDH (1 U/mL) significantly inhibited the loss of ARPE-19 cell viability (Figures 1(c) and 1(d)). These results suggest that serum amine oxidase, oxidative stress, and aldehyde are involved in spermidine-induced ARPE-19 cell death.

### 3.2. Spermidine-Induced Impairment of the Functions of BRB and Retina in Rats

To examine the effect of spermidine on retinal function, including the integrity of the BRB (both outer and inner), BRB permeability [21] and scotopic ERG a- [22] and b-waves [23, 24] were assessed.

**Figure 2:**
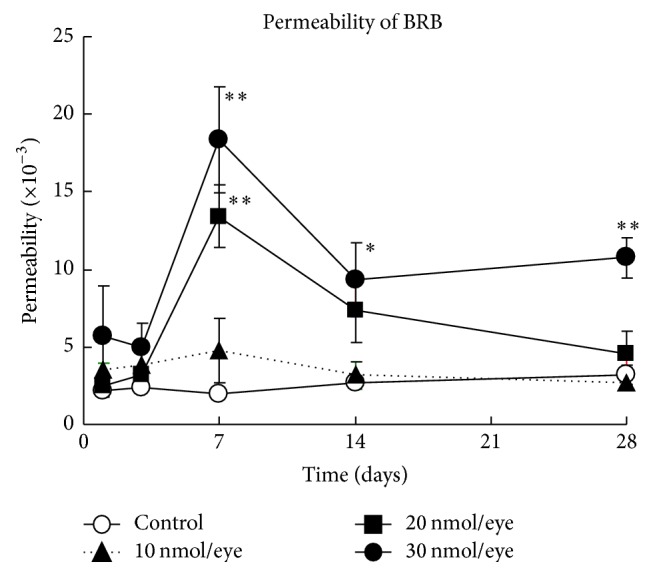
Spermidine-induced hyperpermeability of the blood-retinal barrier (BRB) in rats. BRB permeability was assessed by vitreous fluorophotometry on days 1, 3, 7, 14, and 28 after the intravitreal administration of spermidine. Each value represents the mean ± standard error of the mean of six to eight eyes. ^*∗*^*P* < 0.05, ^*∗∗*^*P* < 0.01 versus control.

Intravitreal administration of spermidine at doses of 20 and 30 nmol/eye significantly increased BRB permeability on day 7 in a dose-dependent manner (Figure 2). By days 14 and 28, however, this spermidine-induced increase in BRB permeability had been attenuated (Figure 2). In contrast, the BRB permeability change brought about by spermidine at 10 nmol/eye underwent recovery to a nonstatistically significant level (Figure 2).

**Figure 3:**
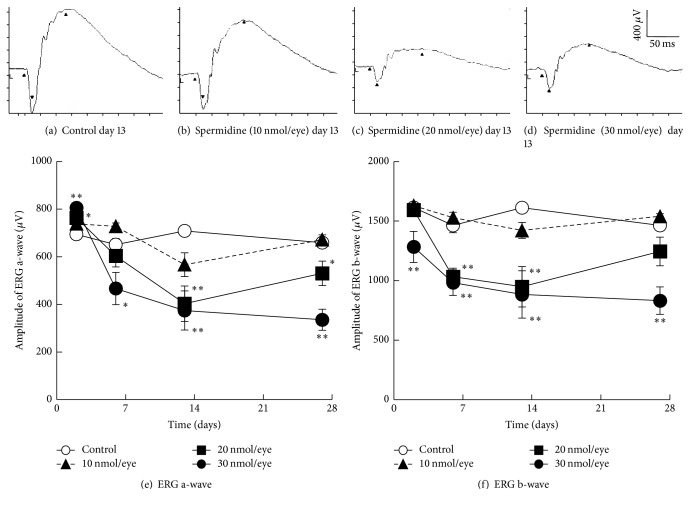
Spermidine-induced impairment of electroretinogram (ERG) a- and b-wave amplitudes in rats. Scotopic ERG a- and b-waves were measured on days 2, 6, 13, and 27 days after the intravitreal administration of spermidine. Traces typical of (a) control, (b) spermidine (10 nmol/eye), (c) spermidine (20 nmol/eye), and (d) spermidine (30 nmol/eye) treatments, 13 days after the injection are shown. Time course data of (e) ERG a-waves and (f) ERG b-waves are shown. Each value represents the mean ± standard error of the mean of seven to eight eyes. ^*∗*^*P* < 0.05 and ^*∗∗*^*P* < 0.01 versus control.

Typical traces of scotopic ERGs recorded from a rat intravitreally injected with either DPBS or spermidine (10–30 nmol/eye) are shown in Figures 3(a)–3(d). Spermidine at 20 and 30 nmol/eye initially increased the ERG a-wave amplitude on day 2; this parameter gradually decreased on days 6 and 13 in a dose-dependent manner (Figure 3(e)). Spermidine at 20 and 30 nmol/eye significantly decreased the ERG b-wave amplitude from day 6 to day 13 and day 2 to day 27, respectively (Figure 3(f)). On day 27, the effect of 20 nmol/eye spermidine was reduced, whereas that of 30 nmol/eye spermidine remained unchanged (Figures 3(e) and 3(f)). In contrast to these two higher doses, 10 nmol/eye spermidine did not have a significant effect on the ERG a- and b-wave amplitudes (Figures 3(e) and 3(f)). These results demonstrate that spermidine at doses of 20 and 30 nmol/eye impairs retinal electrophysiological and barrier functions. In addition, the BRB begins functional recovery on day 14 after administration of 20, but not 30, nmol/eye of spermidine.

### 3.3. Spermidine-Induced RPE and Photoreceptor Cell Degeneration in Rats

**Figure 4:**
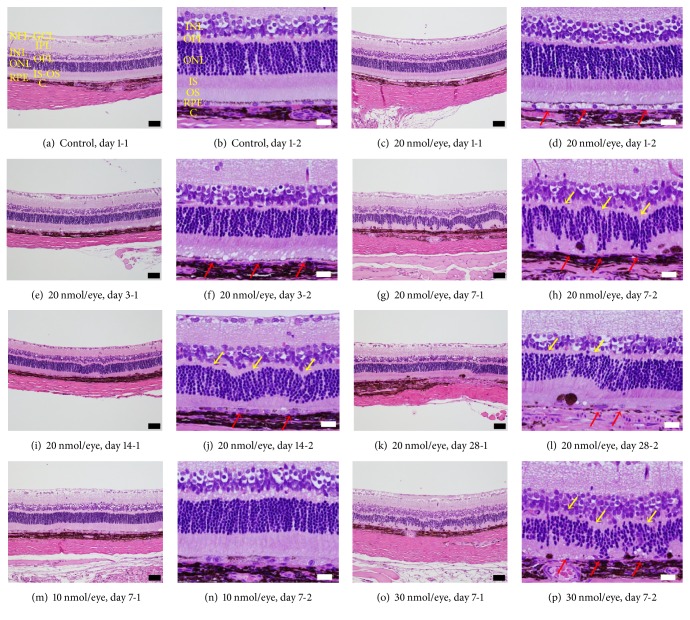
Spermidine-induced degeneration of retinal pigment epithelium (RPE) and photoreceptor cells in rats. Cross-sections of rat eyes were prepared (a, b) 1 day after the administration of DPBS, (c–l) 1, 3, 7, 14, and 28 days after the intravitreal administration of spermidine (20 nmol/eye), and (m–o) 7 days after the injection of spermidine (10 or 30 nmol/eye). Black and white scale bars indicate 50 and 20 *μ*m, respectively. Red arrows in (d) indicate RPE cell vacuolization. Red arrows in (f), (h), and (p) indicate RPE cell degeneration. Red arrows in (j) and (l) indicate RPE cell regeneration. Yellow arrows in (h), (j), (l), and (p) indicate photoreceptor cell degeneration. NFL-GCL: nerve fiber layer and ganglion cell layer. IPL: inner plexiform layer. INL: inner nuclear layer. OPL: outer plexiform layer. ONL: outer nuclear layer. IS: inner segment. OS: outer segment. RPE: retinal pigment epithelium. C: choroid.

Histological analysis revealed that in rats intravitreal injection of 20 nmol/eye spermidine induced RPE cell vacuolization on day 1 (Figure 4(d); red arrows) and RPE cell degeneration on days 3 and 7 (Figures 4(f) and 4(h); red arrows), whereas the intravitreal injection of DPBS did not affect the structure of RPE cells on day 1 (Figures 4(a) and 4(b)). Spermidine at 20 nmol/eye also caused the disruption of photoreceptor outer segments from day 3 to day 28 (Figures 4(f), 4(h), 4(j), and 4(l)) and induced degeneration of the outer nuclear layer from day 7 to day 28 (Figures 4(h), 4(j), and 4(l); yellow arrows). On days 14 and 28, regeneration of RPE morphology was observed (Figures 4(j) and 4(l); red arrows). The spermidine-induced degenerative change of RPE and photoreceptors on day 7 was dose-dependent (Figures 4(h), 4(n), and 4(o)). In addition, spermidine at 20 nmol/eye slightly decreased the thickness of the inner plexiform layer (IPL) and the inner nuclear layer (INL) from days 7 to 28 and days 14 to 28, respectively (Figures 4(g)–4(l)), but spermidine at 10 and 30 nmol/eye did not affect the inner retinal structure on day 7 (Figures 4(m)–4(p)). These results demonstrate that the intravitreal administration of spermidine induced the degeneration of both RPE and photoreceptors. In addition, RPE cell regeneration following treatment with 20 nmol/eye spermidine appeared to begin on day 14.

On the other hand, the intravitreal injection of spermine at 15 nmol/eye induced the atrophy of all retinal layers on day 7 (data not shown). This result suggests that spermidine is a more selective inducer of the degeneration of outer retina than spermine.

**Figure 5:**
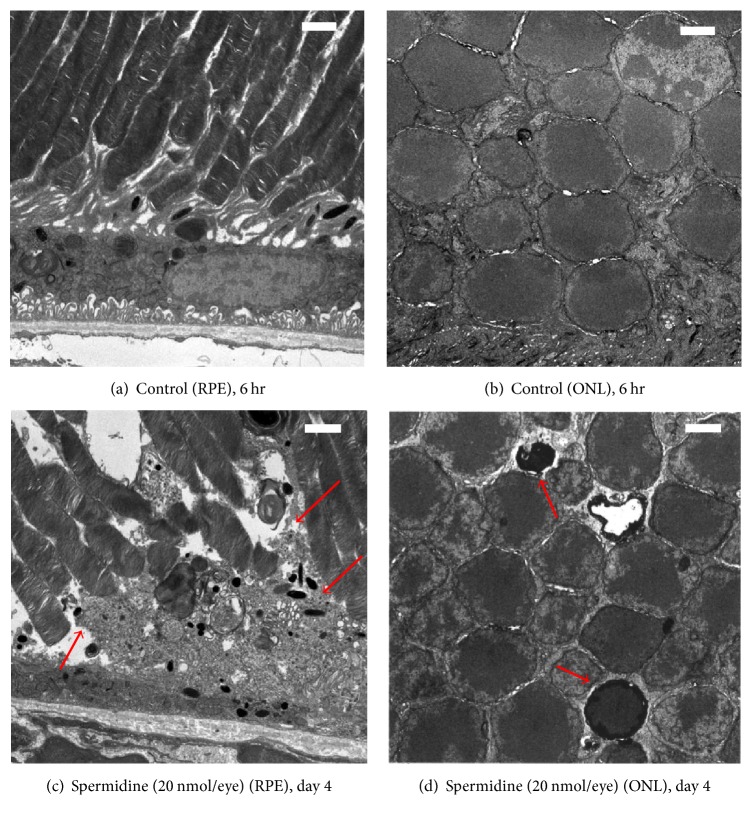
Spermidine-induced ultrastructural changes in the retinal pigment epithelium (RPE) and outer nuclear layer (ONL) of the rat retina. Ultrathin 80–nm sections were prepared from samples fixed 6 hours after the injection of (a, b) DPBS or 4 days after the intravitreal administration of (c, d) spermidine (20 nmol/eye). Scale bars indicate 2 *μ*m. Red arrows in (c) indicate plasma membrane disruption in RPE cells. Red arrows in (d) indicate chromatin condensation in photoreceptor cells.

Ultrastructural analysis revealed that spermidine (20 nmol/eye) disrupted RPE cell plasma membranes on postinjection day 4 (Figure 5(c); red arrows), whereas the intravitreal injection of DPBS did not affect RPE or photoreceptor morphology at 6 hours (Figures 5(a) and 5(b)). In contrast, spermidine did induce chromatin condensation in photoreceptor nuclei by day 4 (Figure 5(d); red arrows). These results support our conclusion that spermidine induces necrosis in RPE cells and apoptosis in photoreceptor cells.

### 3.4. Protective Effects of NAC and ALDH against Spermidine-Induced RPE Degeneration in Rats

**Figure 6:**
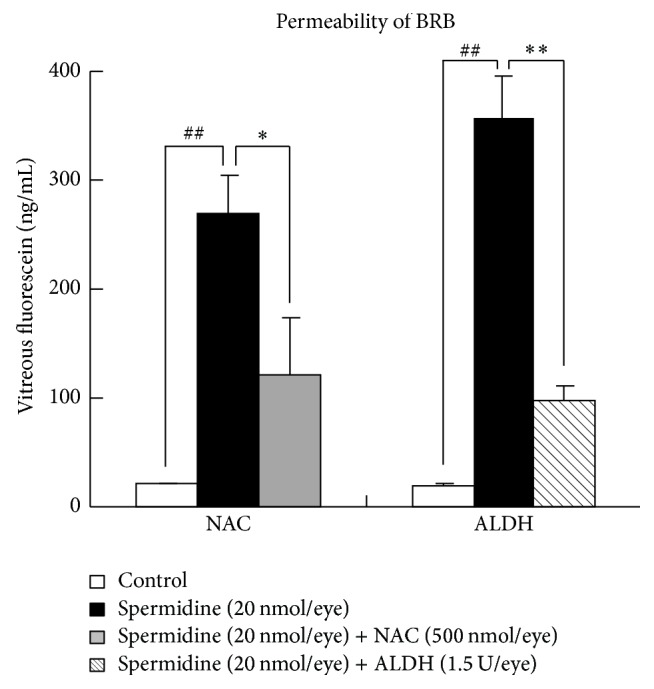
Protective effects of N-acetylcysteine (NAC) and aldehyde dehydrogenase (ALDH) on spermidine-induced hyperpermeability of the blood-retinal barrier (BRB). (a) BRB permeability was assessed by vitreous fluorophotometry 7 days after the intravitreal administration of (a) spermidine (20 nmol/eye) alone, (b) spermidine (20 nmol/eye) plus NAC (500 nmol/eye), and (c) spermidine (20 nmol/eye) plus ALDH (1.5 U/eye). Each column represents a mean ± standard error of the mean of seven to eight eyes. ^##^*P* < 0.01 versus control and ^*∗*^*P* < 0.05 and ^*∗∗*^*P* < 0.01 versus spermidine (20 nmol/eye).

**Figure 7:**
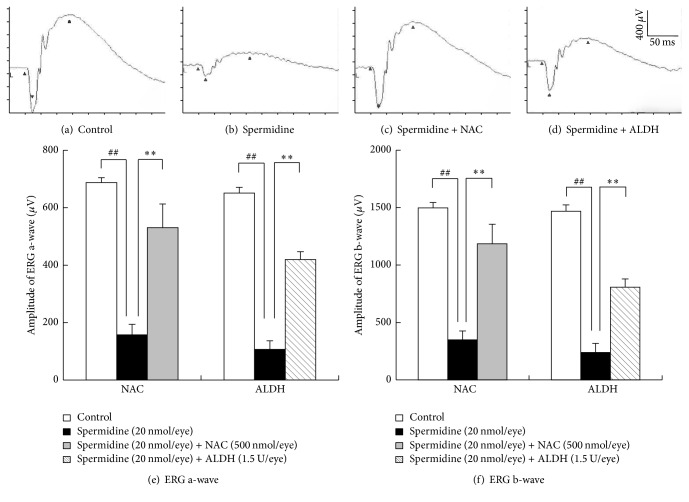
Protective effects of N-acetylcysteine (NAC) and aldehyde dehydrogenase (ALDH) on spermidine-induced impairment of electroretinogram (ERG) a- and b-wave amplitudes. Representative traces are shown of scotopic ERG a- and b-waves measured 13 days after the administration of (a) control, (b) spermidine (20 nmol/eye) alone, (c) spermidine (20 nmol/eye) plus NAC (500 nmol/eye), and (d) spermidine (20 nmol/eye) plus ALDH (1.5 U/eye). The amplitude data for ERG a- and b-waves, from eyes treated as above are depicted in (e) and (f), respectively. Each column represents the mean ± standard error of the mean of seven to eight eyes. ^##^*P* < 0.01 versus control and ^*∗∗*^*P* < 0.01 versus spermidine (20 nmol/eye).

**Figure 8:**
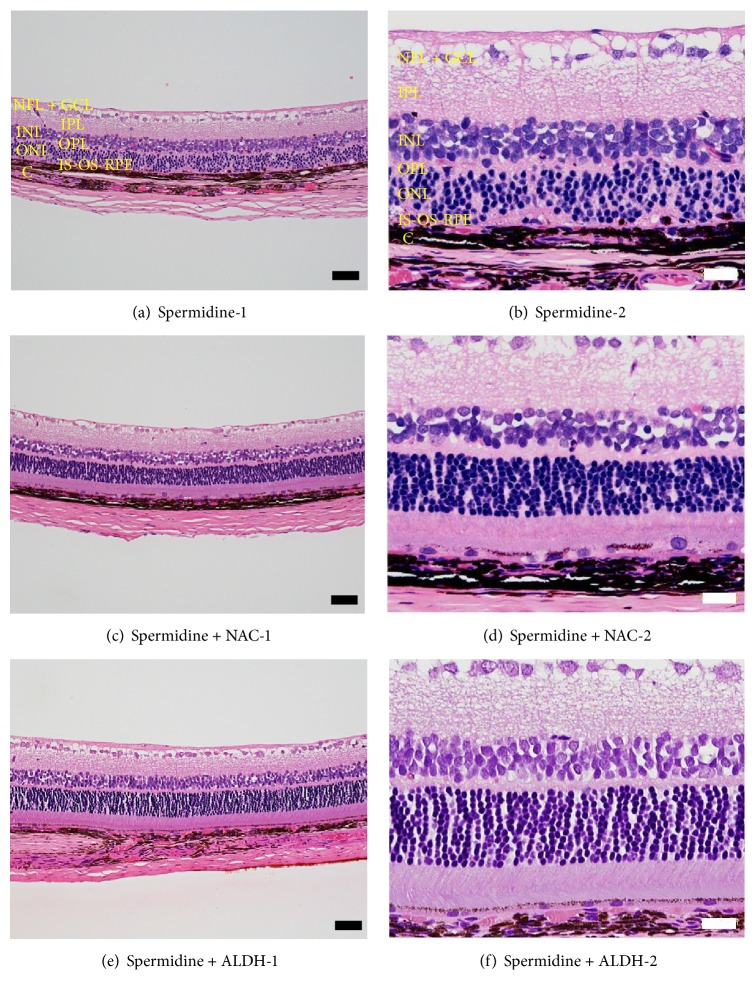
Protective effects of N-acetylcysteine (NAC) and aldehyde dehydrogenase (ALDH) on spermidine-induced degeneration of the retinal pigment epithelium (RPE) and photoreceptors in rats. Cross-sections of rat eyes were prepared 13 days after the intravitreal injection of (a, b) spermidine (20 nmol/eye) alone, (c, d) spermidine (20 nmol/eye) plus NAC (500 nmol/eye), and (e, f) spermidine (20 nmol/eye) plus ALDH (1.5 U/eye). Black and white scale bars indicate 50 and 20 *μ*m, respectively. NFL-GCL: nerve fiber layer and ganglion cell layer. IPL: inner plexiform layer. INL: inner nuclear layer. OPL: outer plexiform layer. ONL: outer nuclear layer. IS-OS-RPE: inner segment, outer segment, and retinal pigment epithelium. C: choroid.

**Figure 9:**
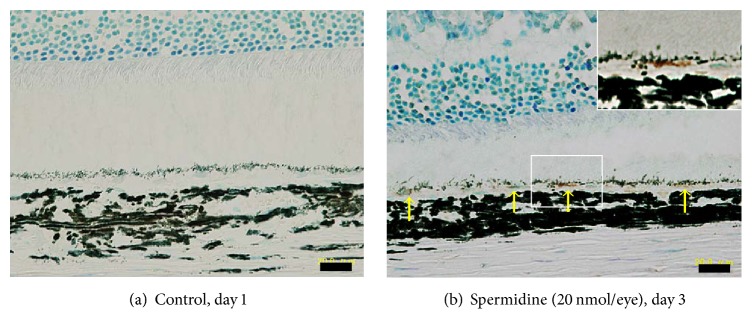
Immunohistochemistry of acrolein in spermidine-treated rat retina. Immunohistochemical localization of acrolein was performed using cross-sections of rat eyes which were prepared (a) 1 day and (b) 3 days after the intravitreal injection of DPBS or spermidine (20 nmol/eye). Scale bars indicate 20 *μ*m. Inserted photograph in (b) is an enlarged image of the same slide.

To clarify the involvement of oxidative stress and aldehyde toxicity in spermidine-induced RPE degeneration in this rat model, the effects of NAC and ALDH were examined. Both NAC (500 nmol/eye) and ALDH (1.5 U/eye) significantly inhibited spermidine-induced hyperpermeability of the BRB on day 7 (Figure 6) and impairment of the ERG a- and b-wave amplitudes on day 13 (Figure 7). Moreover, NAC and ALDH prominently inhibited the spermidine-induced degeneration of RPE and photoreceptors previously observed by day 13 (Figure 8). In addition, the immunodetection of acrolein-modified proteins (which are highly reactive and longer-lived than free acrolein [25]) was observed only within the RPE of a spermidine injected eye (Figure 9(b)). These results, which are consistent with those of our in vitro findings, suggest that oxidative stress and toxic aldehyde byproducts are involved in spermidine-induced RPE degeneration in rats.

## 4. Discussion

A major finding of the present study is that the intravitreal administration of spermidine induced the dysfunction and death of RPE cells in association with the degeneration of photoreceptors in rats. Although our results do not rule out spermidine also affecting the structure and function of the inner retina under our experimental conditions, the most prominent histological changes induced by this molecule were observed in the outer retina. This is, to our knowledge, a novel animal model of RPE degeneration mediated by spermidine, and more specifically by oxidation products of spermidine.

To elucidate the mechanisms underlying spermidine-induced RPE cell death, we examined the effects of various polyamine oxidation pathway inhibitors on spermidine-induced RPE cell death in both in vitro and in vivo studies. Aminoguanidine and pentamidine (amine oxidase inhibitors), as well as NAC (an antioxidant) and ALDH (an aldehyde-quenching enzyme), suppressed spermidine-induced ARPE-19 cell death in our in vitro study, and the latter two agents also ameliorated RPE dysfunction and death in our in vivo experiments. In addition, the immunolocalization of acrolein-modified macromolecules was observed in RPE cells of a spermidine-treated eye. These results are consistent with previous studies which showed that amine oxidase inhibitors, NAC, and/or ALDH suppressed the polyamine-induced cell death of cancer cell lines [16, 17] and microglia [18]. NAC is believed to suppress oxidative stress resulting from either polyamine oxidation [26] and/or as a result of increased production of acrolein [18, 27]. ALDH is an aldehyde-quenching enzyme and known to metabolize acrolein [28]. Therefore, these results and previous evidence suggest that spermidine-induced RPE cell death is mediated by hydrogen peroxide and the toxic aldehyde acrolein, both generated by spermidine oxidases both in vitro and in vivo. Importantly, both hydrogen peroxide and acrolein have been reported to induce the oxidative stress-mediated death of ARPE-19 cells [29–31].

In this study, the intravitreal administration of spermidine induced hyperpermeability of the BRB in rats, as assessed by vitreous fluorophotometry. The BRB is comprised of inner and outer components, corresponding to intercellular tight junctions between retinal endothelial, and RPE cells, respectively [32], and the breakdown of either or both the inner and/or the outer BRB may contribute to the observed retinal hyperpermeability. Based on the results of our histological analysis, the disruption of outer BRB contributes at least in part to the hyperpermeability of BRB which we documented in spermidine-treated animals. However, the added contribution of changes in the inner BRB in spermidine-treated eyes cannot at this point be ruled out. Further study is needed to elucidate this.

We found that the intravitreal administration of spermidine had a biphasic effect on the functions of photoreceptor cells, as determined through an evaluation of ERG a-wave amplification in rats. The reason for this spermidine-induced initial increase in ERG a-wave amplitudes on day 2 is unclear, although a similar early enhancement effect on ERG a-waves was reported in another RPE degeneration model [33, 34]. Therefore, further studies are needed to elucidate this phenomenon. On the other hand, the later spermidine-induced decrease in ERG a-wave amplitude from days 6 to 27 is attributable to the impaired function and, in some cases, loss of photoreceptor cells because spermidine induced disruption of photoreceptor outer segments from day 3 to day 28, and outer nuclear layer degeneration was evident from day 7 to day 28.

Our histological analysis revealed that spermidine initially induced RPE cell damage and degeneration, whereas photoreceptor cell death was a secondary outcome. This phenomenon could be explained by the loss of support provided by RPE cells to photoreceptor cells [1]. In fact, similar changes were reported in other animal models of RPE degeneration, including sodium iodate [7] and ornithine-induced models [10] and the ornithine delta-aminotransferase deficient mouse [11], and a similar scenario also has been invoked as a mechanism in human dry AMD [2, 3] and gyrate atrophy [4].

Spermidine is suggested to impair the function of inner retinal elements such as bipolar and Müller cells, since spermidine at 20 and 30 nmol/eye decreased the ERG b-wave amplitude, without decreasing ERG a-wave amplitude, on day 6 and day 2 following injection, respectively. Since spermidine is reported to block the potassium channels of retinal Müller cells [35], spermidine may suppress the function of Müller cells without spermidine oxidation. In addition, the slight degeneration of the inner retina by spermidine might also contribute to this effect, since spermidine at 20 nmol/eye slightly decreased the thickness of IPL and INL in this study. However, we think that further precise studies are needed to determine whether this slight histological change in the inner retina induced by spermidine is a significant degeneration or an artifact, because photographs of HE-stained sections were not taken at exactly the same position of the eye for all histological samples, and there was no evidence of the dose-dependency of spermidine relative to this change.

It is important to elucidate the mechanism underlying the prominent degenerative effect of spermidine on RPE, since excess polyamines are known to induce cell death of neurons [36] and glia [18, 37] as well as RPE [15]. In fact, intravitreal administration of spermine (15 nmol/eye), a stronger inducer of cell death than spermidine [15, 37], in our hands induced the atrophy of all retinal layers. Although there were different degrees of retinal degeneration induced by spermine and spermidine, the changes observed in the outer retina indicate that RPE cells are more directly sensitive to the toxic effects of polyamines than other retinal cells. Moreover, our immunohistochemical analysis showed that acrolein was detected only in RPE cells following spermidine treatment. This result suggests that the higher production of acrolein due to the higher activity of spermidine oxidation in RPE than other retinal cells may be one of the reasons for the higher sensitivity of RPE cells to the more localized, immediate toxic effect of spermidine.

We found that spermidine (20 nmol/eye)-induced RPE degeneration was transient and observed RPE recovery on days 14 and 28. One recent study showed that rat RPE cells could enter the cell cycle and complete cellular division [38]. In fact, several reports have shown that RPE regeneration occurs after low-dose but not high-dose sodium iodate-induced RPE degeneration in mice [39, 40]. Therefore, RPE regeneration is believed to occur after modest RPE degeneration.

It is noteworthy that RPE cells and photoreceptors experienced different modes of cell death in response to spermidine-induced retinal degeneration. In our hands, spermidine appeared to induce RPE cell death by necrosis following disruption of the plasma membrane in these cells, whereas spermidine has been reported previously to induce cell death by both necrosis and apoptosis [11, 13]. The mode of spermidine-induced cell death might depend on experimental conditions such as the cell type and spermidine concentration. According to a recent review article [41], although there is still some controversy regarding the mechanism(s) of RPE cell death in human dry AMD, ultrastructural and histopathological studies support necrosis as the major mechanism of RPE cell death in dry AMD. Although there are no reports about the mode of RPE cell death in human gyrate atrophy with hyperornithinemia, necrosis of RPE cell was observed in ornithine-induced retinopathy in rats [10]. Therefore, there are likely to be some equivalent RPE cell death pathways between the spermidine model presented here and human dry AMD and gyrate atrophy. In contrast, the secondary loss of photoreceptor cells overlying damaged RPE cells might be caused by apoptosis, as spermidine caused chromatin condensation in photoreceptor cells. This phenomenon is therefore not unique to our model and might be common to other types of retinal degeneration, given that similar results were reported in the contexts of sodium iodate-induced RPE degeneration in mice [42], ornithine-induced retinopathy in rats [10], and human dry AMD [43].

In contrast to our study, Noro et al. showed that spermidine has a protective effect on retinal ganglion cells in mouse models of optic nerve injury and normal tension glaucoma [44, 45]. In Noro's reports, spermidine was administrated by drinking water at a concentration of 30 mM [44, 45]. The effective retinal concentration of spermidine by administration via drinking water containing this agent would be expected to be much lower than what we obtained by intravitreal injection of this compound (1–3 mM). At different concentrations in the retina, spermidine indeed may demonstrate pleiotropic effects. At an optimal, permissive concentration, spermidine is known to be necessary for RPE proliferation [13], but excess spermidine is shown to cause RPE cell death [15]. Therefore, the contrasting effects of spermidine between Noro's studies and our own may be explained by the different concentrations of spermidine effectively delivered to the retina.

The clinical significance of spermidine oxidation in the posterior segment of the eye is currently unclear, as increased spermidine levels have not been reported in human diseases involving RPE degeneration. Further studies to evaluate spermidine concentrations and amine oxidase activity in patients with dry AMD and gyrate atrophy are needed to clarify this point. However, the phenotype of spermidine-induced retinal degeneration described herein shares some common features with the phenotypes of dry AMD [2, 3] and gyrate atrophy [4]. Additionally, the involvement of oxidative stress in this model has been proposed as a general mechanism of RPE degeneration in dry AMD [5]. Moreover, spermidine might be involved in the pathogenesis of gyrate atrophy with hyperornithinemia, as spermidine is an ornithine metabolite [7]. Therefore, an animal model of spermidine-induced RPE degeneration could be potentially useful as a model of both dry AMD and gyrate atrophy.

In conclusion, the results of this study demonstrate that the intravitreal administration of spermidine induces rat RPE cell dysfunction and death, leading to photoreceptor cell degeneration. These effects of spermidine are thought to be mediated by oxidative stress and a toxic aldehyde generated by amine oxidase as a consequence of spermidine oxidation. Although the effect of spermidine on the inner retina was not fully clarified, following further validation, this novel animal model will likely be not only useful for elucidating the pathophysiology of dry AMD and gyrate atrophy, but also useful for screening potential therapies for these diseases.
